# A novel functional variant in 8q24 is associated with regulation of prostate stem cell antigen (*PSCA*) gene expression and bladder cancer risk

**DOI:** 10.1186/gb-2011-12-s1-p4

**Published:** 2011-09-19

**Authors:** Yi-Ping Fu, Indu Kohaar, Adam Mumy, Wei Tang, Brian Muchmore, Patricia Porter-Gill, Luyang Liu, Jonine Figueroa, Montserrat Garcia-Closas, Dalsu Baris, Mark Purdue, Michael Thun, Demetrius Albanes, Nuria Malats, Francisco X Real, Manolis Kogevinas, Alison Johnson, Molly Schwenn, Stephen Chanock, Nathaniel Rothman, Debra Silverman, Ludmila Prokunina-Olsson

**Affiliations:** 1 Laboratory of Translationa Genomics, Division of Cancer Epidemiology and Genetics, National Cancer Institute, National Institutes of Health, Bethesda, MB 20892, USA; 2 Division of Cancer Epidemiology and Genetics, National Cancer Institute, National Institutes of Health, Bethesda, MD 20892, USA; 3 Epidemiology Research Program, American Cancer Society Atlanta, GA 30303, USA; 4 Spanish National Cancer Research Center, Madrid 28029, Spain; 5 Centre for Research in Environmental Epidemiology, Barcelona 08003, Spain; 6 Vermont Cancer Registry Burlington, VT 05401, USA; 7 Maine Cancer Registry Augusta, ME 04333, USA

## Background

Recent genome-wide association studies (GWAS) have identified allele T of a single nucleotide polymorphism (SNP), rs2294008, in the prostate stem cell antigen (*PSCA*) gene as a risk factor for bladder cancer [1,2]. In the present study, we aimed to find additional disease-associated SNPs in the *PSCA* region and to explore their possible molecular function.

## Methods

Based on information from the 1000 Genomes and HapMap 3 projects, we performed imputation analysis on 3,532 bladder cancer cases and 5,120 healthy controls of European ancestry from the stage 1 bladder cancer GWAS, within ±100 kb of the region flanking the GWAS signal, rs2294008. The average allele dosage and best-guess genotypes were estimated and tested for association between SNP variants and bladder cancer risk by using unconditional logistic regression. Functional follow-up studies included RNA sequencing in normal and tumor bladder samples and electrophoretic mobility shift assays to examine the potentially altered DNA-protein interactions for SNPs of interest.

## Results

A total of 639 imputed and 37 genotyped SNPs within ±100 kb of the region of the original GWAS signal were tested for genetic association with bladder cancer. In these stage 1 GWAS samples, the SNP rs2294008 had a per-allele odds ratio (OR) of 1.09 (95% confidence interval (CI) = 1.02 to 1.16, *P* = 6.93 × 10^–4^). Multivariable logistic regression analysis adjusted for the study center, age, gender, smoking status and rs2294008 genotype revealed a novel associated variant, rs2978974 (OR = 1.11, 95% CI = 1.04 to 1.19, *P* = 1.62 × 10^–3^). There was low linkage disequilibrium between rs2978974 and the original GWAS signal, rs2294008 (D⊠ = 0.19, *r*^2^ = 0.02). Only individuals carrying the risk variant of both SNPs had an increased risk of bladder cancer (OR = 1.24, 95% CI = 1.13 to 1.35, *P* = 4.69 × 10^–6^) and not individuals who carried a risk variant of only one of the SNPs (*P* > 0.05). Stratified analysis suggested that this compound effect of rs2294008 and rs2978974 was more significant in males (OR = 1.27, *P* = 2.80 × 10^–6^) than in females (OR = 1.08, *P* = 0.52).

rs2978974 resides 10 kb upstream of rs2294008, is marked by an H3K4me3 signal and is in the vicinity of an androgen-receptor-binding site. Using RNA sequencing of bladder samples, we showed that rs2978974 is located within an alternative, untranslated first exon of *PSCA.* Using the electrophoretic mobility shift assay with nuclear proteins from LNCaP and HeLa cells, we observed that the non-risk-associated allele (G) of rs2978974, but not the risk allele (A), could bind to ELK1, a protein belonging to the ETS family of transcription factors.

## Conclusions

We identified a SNP, rs2978974, in the *PSCA* region as a novel marker for bladder cancer susceptibility. There was a compound effect in carriers of both the rs2294008 and rs2978974 risk variants. The functional relevance of rs2978974 might be related to the loss of ELK1 regulation by the risk allele (A) and differential regulation of *PSCA* mRNA expression.
